# Mapping the membrane proteome of anaerobic gut fungi identifies a wealth of carbohydrate binding proteins and transporters

**DOI:** 10.1186/s12934-016-0611-7

**Published:** 2016-12-20

**Authors:** Susanna Seppälä, Kevin V. Solomon, Sean P. Gilmore, John K. Henske, Michelle A. O’Malley

**Affiliations:** 1 Novo Nordisk Foundation Center for Biosustainability, Technical University of Denmark, Kemitorvet Bygning 220, 2800 Kgs. Lyngby, Denmark; 2 Department of Chemical Engineering, University of California, Santa Barbara, CA 93106 USA; 3 Agricultural and Biological Engineering, Purdue University, West Lafayette, IN 47907 USA

**Keywords:** Microbial engineering, Membrane proteins, Anaerobic fungi, Carbohydrate binding proteins, Lignocellulose

## Abstract

**Background:**

Engineered cell factories that convert biomass into value-added compounds are emerging as a timely alternative to petroleum-based industries. Although often overlooked, integral membrane proteins such as solute transporters are pivotal for engineering efficient microbial chassis. Anaerobic gut fungi, adapted to degrade raw plant biomass in the intestines of herbivores, are a potential source of valuable transporters for biotechnology, yet very little is known about the membrane constituents of these non-conventional organisms. Here, we mined the transcriptome of three recently isolated strains of anaerobic fungi to identify membrane proteins responsible for sensing and transporting biomass hydrolysates within a competitive and rather extreme environment.

**Results:**

Using sequence analyses and homology, we identified membrane protein-coding sequences from assembled transcriptomes from three strains of anaerobic gut fungi: *Neocallimastix californiae*, *Anaeromyces robustus*, and *Piromyces finnis*. We identified nearly 2000 transporter components: about half of these are involved in the general secretory pathway and intracellular sorting of proteins; the rest are predicted to be small-solute transporters. Unexpectedly, we found a number of putative sugar binding proteins that are associated with prokaryotic uptake systems; and approximately 100 class C G-protein coupled receptors (GPCRs) with non-canonical putative sugar binding domains.

**Conclusions:**

We report the first comprehensive characterization of the membrane protein machinery of biotechnologically relevant anaerobic gut fungi. Apart from identifying conserved machinery for protein sorting and secretion, we identify a large number of putative solute transporters that are of interest for biotechnological applications. Notably, our data suggests that the fungi display a plethora of carbohydrate binding domains at their surface, perhaps as a means to sense and sequester some of the sugars that their biomass degrading, extracellular enzymes produce.

**Electronic supplementary material:**

The online version of this article (doi:10.1186/s12934-016-0611-7) contains supplementary material, which is available to authorized users.

## Background

The design and construction of microbial cell factories is often focused on the engineering of intracellular enzymes and pathways, and the role of membrane-embedded proteins is often overlooked. It is nevertheless becoming increasingly clear that integral membrane proteins, in particular transporters, are critical for the performance and stability of microbial production strains [1–4]. Membrane-embedded transport proteins mediate the cellular uptake and extrusion of a wide diversity of solutes. As a consequence, adding or engineering a suitable uptake system into a production strain may greatly enhance substrate utilization and flux towards product [5–7]. Likewise, secretion systems increase flux, resolve toxicity-caused limitations, and facilitate product purification by secretion of the desired product to the extracellular environment [8–10].

Besides introducing and engineering known transport proteins for biotechnological applications, there is a critical need to identify novel transporters from the wealth of sequence data that we are currently amassing from nature [11]. Fortunately, integral membrane proteins are typically easy to predict from primary sequence data, as they contain one or more stretches of ~20 hydrophobic amino acid residues (transmembrane segments). Similarly, secreted proteins that contain a hydrophobic, amino-terminal cleavable signal peptide can also be identified using this approach [12–15]. Using sequence analyses and homology, putative membrane proteins may be sorted into functional classes such as receptor proteins or solute transporters, to serve as guidelines for downstream experimental targeting and characterization [16, 17].

To increase the known repertoire of transporters that are of particular interest for cell factory engineering, it is inviting to look at the membrane proteins from organisms that nature has adapted for food sources that are out of reach for conventional microbes. One such example is the anaerobic gut fungi that inhabit the intestines of herbivores such as horses and sheep, where they secrete powerful cellulases and other saccharolytic enzymes that break down recalcitrant plant biomass into digestible sugars [18–21]. A variety of hydrolyzed sugars and low molecular weight cellodextrins are both sensed and transported across fungal bilayers. Whereas their tremendous biotechnological potential is unquestionable, isolation and cultivation of the gut fungi under laboratory conditions has proven challenging and only a relatively small number of strains have been isolated to date. Moreover, very little is known about the membranes and membrane proteins of these early branching fungi, and the extreme AT-richness of their genomes have precluded high quality genomic data from being obtained [22, 23].

Here, we mined transcriptomic data collected from three recently isolated strains of anaerobic fungi, *Neocallimastix californiae*, *Anaeromyces robustus* and *Piromyces finnis*, for integral membrane proteins that would shed light on the physiology of these unusual microorganisms. In particular, we sought to characterize the membrane-bound machinery that underlies their remarkable ability to survive and persist in the competitive, biomass-rich environment of the herbivore gut. We hypothesized that apart from the secreted biomass-degrading enzymes, the fungi possess membrane-embedded transporters and receptors that support the lignocellulolytic lifestyle of the fungi and confer an ecological or evolutionary advantage [24, 25]. Importantly, the transporters and receptors that we identify have the potential to advance metabolic engineering efforts for biomass utilization and conversion in model microbes [19]. Overall, this serves as the first comprehensive study of the membrane protein components within anaerobic gut fungi, providing deeper insight into the physiology of these understudied organisms, and a wealth of transporters and receptors that can be further adopted for strain engineering.

## Results and discussion

### Integral membrane proteins in anaerobic gut fungi: a birds-eye view

**Fig. 1:**
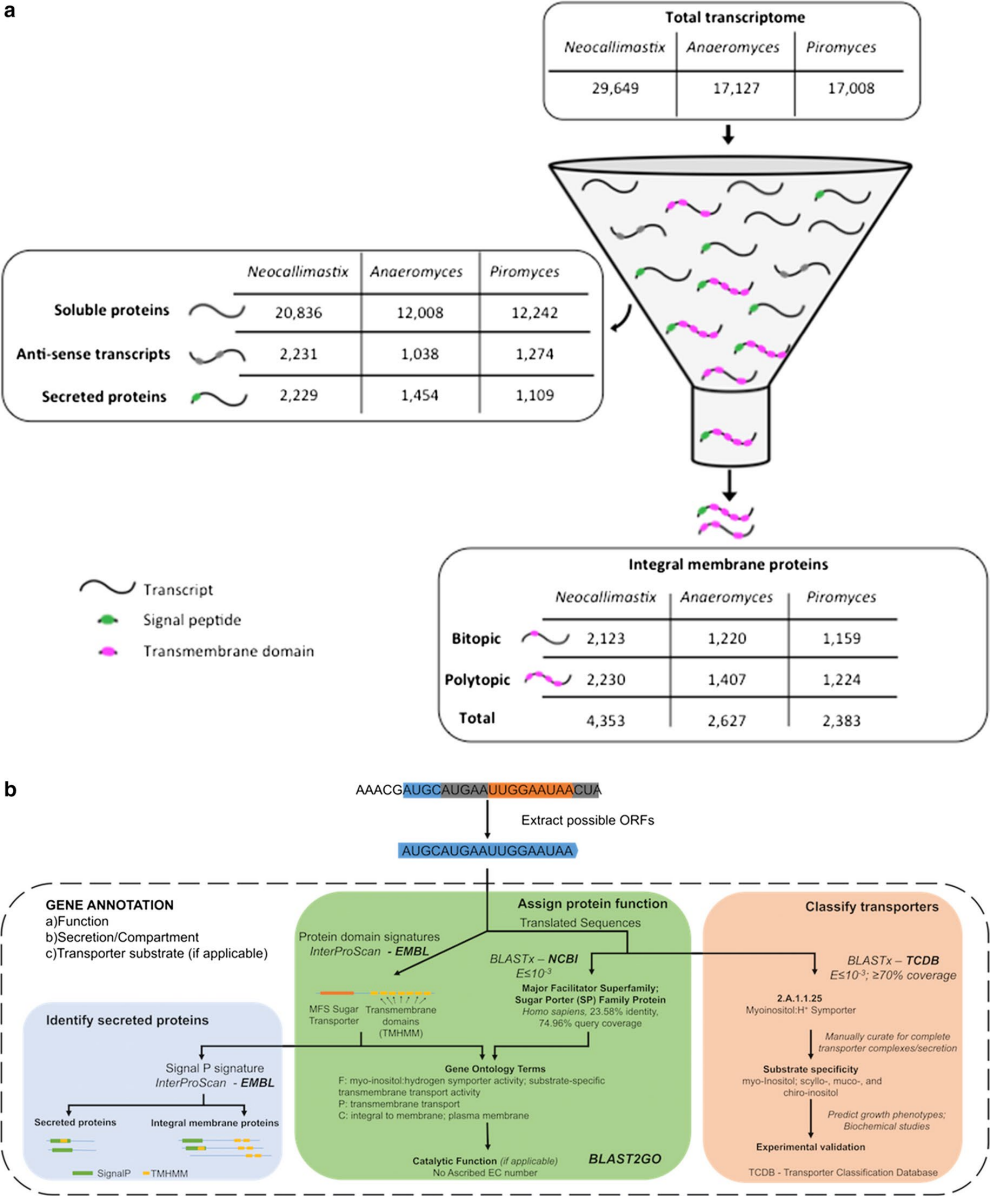
Integral membrane proteins are identified from gut fungal transcriptomes using bioinformatics filtering. **a** Displays a quantitative ‘funneling process’, where the total transcriptome is reduced to the membrane protein component by filtering the predicted soluble proteins, antisense transcripts, and extracellularly secreted proteins. **b** Demonstrates the pipeline used for protein annotation. All possible ORFs are extracted from the assembled transcripts, and protein annotations, gene ontology (GO) terms, and enzyme commission (EC) numbers are obtained by aligning the ORFs to the NCBI database (E ≤10^−3^) with BLASTx and comparing the ORFs to the EMBL database with the InterProScan tool. InterProScan utilizes SignalP and TMHMM to predict ER targeting signal peptides and transmembrane domains. Finally, the ORFs are aligned to the TCDB database to identify possible transporters and predict transporter substrates

Recently, three novel strains of anaerobic gut fungi were isolated from animal feces: *Neocallimastix californiae* (*N. californiae*) from goat, *Anaeromyces robustus* (*A. robustus*) from sheep, and *Piromyces finnis* (*P. finnis*) from horse [26]. To assemble a complete transcriptome, RNA was collected from each strain grown on a number of different representative substrates ranging from insoluble plant material and cellulose to soluble carbon sources such as cellobiose and glucose [19]. Here, we identified secreted and integral membrane proteins from over 60,000 transcripts using complementary bioinformatics approaches. As is shown in Fig. 1, about 20% of assembled transcripts in each fungal strain encode proteins that have a predicted signal peptide and/or transmembrane segments [15, 27]. About a third of the trafficked proteins are predicted to be completely secreted to the extracellular environment; among these are cellulases, glucosidases and proteases that allow the fungi to degrade plant material extracellularly into soluble sugars [19, 28]. The remaining two thirds of the trafficked proteins have at least one predicted non-cleaved transmembrane segment, and as such they are likely integral membrane proteins: there are 4353 transcripts encoding putative membrane proteins in *N. californiae*, 2627 membrane protein transcripts in *A. robustus*, and 2383 membrane protein transcripts in *P. finnis* (Fig. 1). Almost half of these proteins are predicted to have only one transmembrane segment, *i.e.* they are bitopic, and these are displayed separately as these proteins may be cleaved and released to the extracellular environment [15].

**Fig. 2:**
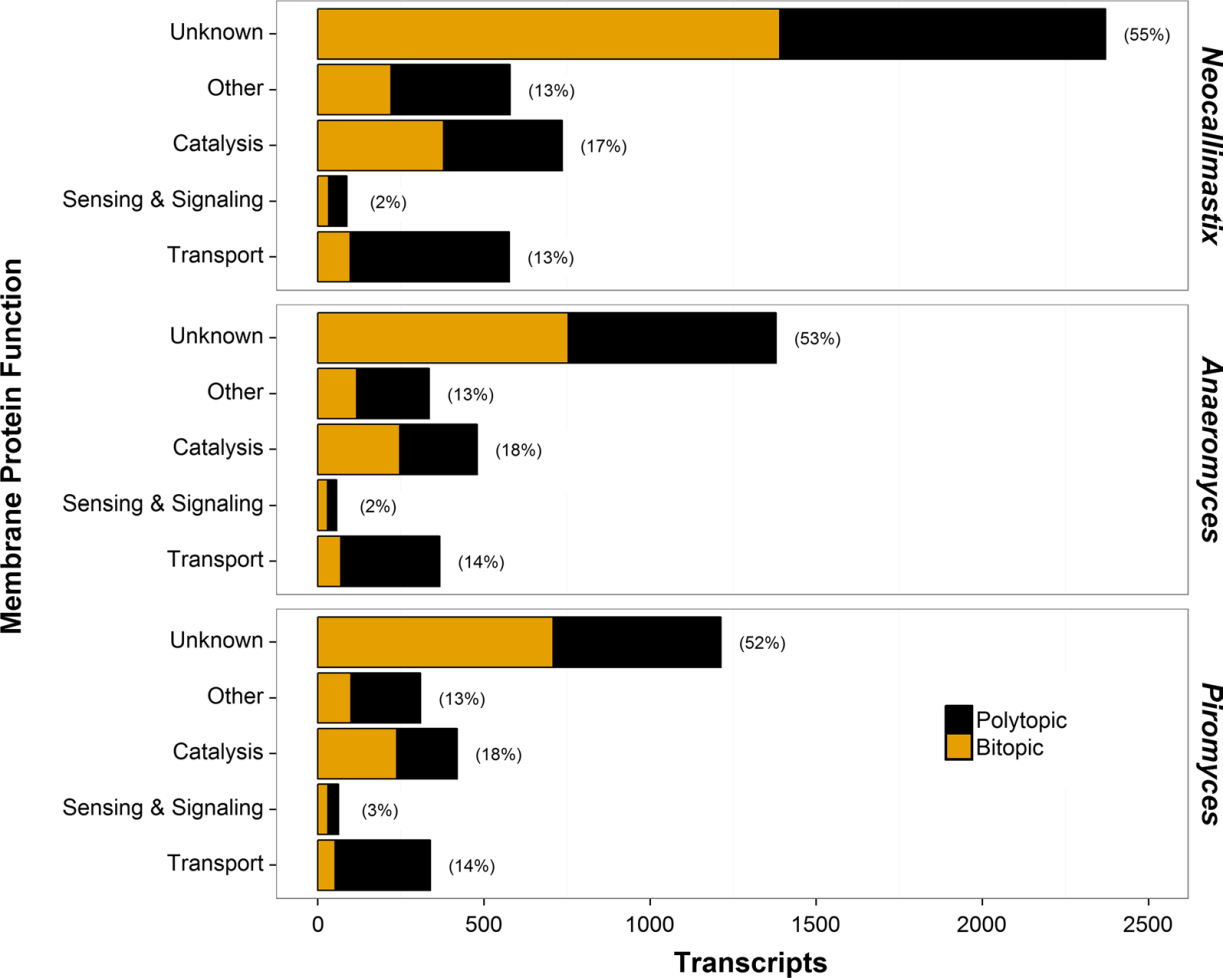
Putative functions of integral membrane proteins in three strains of anaerobic gut fungi as classified by gene ontology (GO). The strains in this study represent three of seven currently acknowledged genera: *Neocallimastix californiae*, *Anaeromyces robustus* and *Piromyces finnis*. Integral membrane protein candidates were binned into one of five functional categories as described in the methods section. The percentages show how many of the predicted integral membrane proteins in each strain falls within a given category

As shown in Fig. 2, gene ontology (GO) annotations suggest that at least a third of the membrane proteins in each fungal strain are involved in transport, sensing, signaling or catalysis [29]. Within these groups are pumps and channels for diverse solutes, peptides and proteins; GPCRs and associated factors; and proteins with catalytic activity such as cellulases, chitinases, glucosidases and glycosyl transferases. Many proteins have more than one GO-term and thus more than one putative function. Here each transcript was counted only once, and as a consequence the assignment of these classes is not exhaustive. For example, manual inspection reveals that a number of the ‘Catalysis’ proteins (proteins that have a GO-term ending with ‘-ase’) are transporters that hydrolyze ATP as part of the transport process, and similarly Receptor Tyrosine Kinases are known to have major functions in cellular signaling and sensing [30, 31]. Likewise, although the ‘Other’ category contains proteins that have ‘other functions’ such as adhesion proteins and chaperones, this class also contains a number of transporters and receptors. Overall, initial bioinformatics funneling and sorting of the membrane proteome reveals the expected machinery for a microbe that deconstructs biomass and catabolizes hydrolyzed byproducts. Notably, around half of the predicted membrane proteins do not have a GO-annotation. This is likely because the relatively low natural abundance and amphiphilic nature of membrane proteins renders their characterization and classification challenging, and thus they are poorly represented in sequence databases. In particular, small membrane proteins have received much less attention than their larger counterparts, and consequently many of the bitopic membrane proteins fall into the ‘Unknown’ category [32–34]. In addition to these limitations, it is important to note that no high-quality genomic sequences exist to describe the early-branching fungi, and only roughly 30% of each transcriptome can be annotated through comparison to the NCBI databank [19, 35].

### Transporters in the anaerobic gut fungi

To gain a deeper understanding of the underlying systems that permit the gut fungi to mediate transport of sugars and other metabolites, we aligned assembled transcripts to the transporter classification database (TCDB) using BLASTx [17, 36, 37]. TCDB is a manually curated database that organizes proteins according to function and phylogeny. In TCDB, each transport system receives a five-tiered identity tag to describe its familial relationship and function, and this gives us the opportunity to sort the transporter proteins at finer resolution. As many transporters contain subunits that are only peripherally associated with the membrane, we included all transcripts in this analysis, regardless of whether the proteins were predicted to have transmembrane segments or not. This inclusive approach also allowed us to identify putative beta-barrel membrane proteins that are present in the outer membranes of mitochondria and plastids, and that TMHMM fails to identify since they lack the canonical alpha-helical stretches of hydrophobic amino acid residues [38, 39]. To increase confidence in transporter predictions, we applied a stringent 70% coverage criterion, where 70% of the query sequence must match the subject sequence, and *vice versa*, with an E- value less than 10^−3^.

**Fig. 3:**
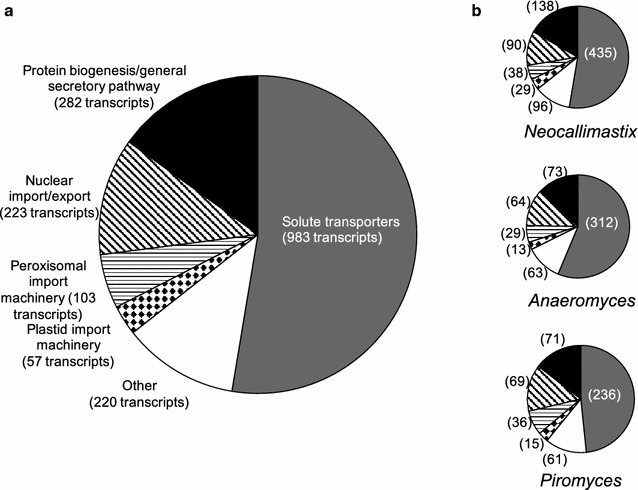
Putative functions of fungal transporters based on transporter classification data base (TCDB) analysis. **a** 1868 fungal transporter components from three gut fungal strains were sorted based on TCDB homology using a stringent 70% coverage criterion. The major functional transporter categories are: solute transport, protein biogenesis/general secretory pathway, nuclear import/export, peroxisomal import machinery, and import into plastids (hydrogenosomes). The “Other” category contains accessory factors and incompletely characterized transport systems. **b** Shows the distribution of the functional categories in the three gut fungal strains. Total number of transcripts encoding transporter components in *Neocallimastix*: 826 transcripts; *Anaeromyces*: 554 transcripts; *Piromyces*: 488 transcripts. The number of transcripts in the different categories is shown in *brackets*

As shown in Fig. 3, using these stringent criteria, we identified 826 transcripts in *Neocallimastix*; 554 transcripts in *Anaeromyces*; and 488 transcripts in *Piromyces* that encode putative transporter system components. For engineering purposes, it is worth noting that the minimal functional unit of many solute carriers is a single polypeptide, whereas other transporter systems are multi-subunit complexes such as the large nuclear pore complex (multiple copies of ~30 different subunits) [40], meaning that the actual numbers of *complete* transport systems is somewhat smaller than that shown here. Also, it is important to take into account the energy requirements of the transporter, that is, whether they are passive channels or use e.g. ATP hydrolysis or an ion gradient to pump solutes across the membrane (Additional file 1: Figure S1). Notably, the placement of a protein in a certain category is not always unequivocal; e.g. here we have placed nucleotide-sugar transporters in the solute transporter category, although most of these are likely localized to the ER and Golgi membranes and their function is in protein biogenesis, as many proteins are expected to be glycosylated while they progress through the secretory pathway [35]. Nevertheless, it is clear that all three strains have a number of conserved transport systems that are involved in protein biogenesis and intracellular sorting, and that approximately half of all transport systems in all three strains are involved in transmembrane translocation of a range of small solutes. These systems are described in more detail below.

### Proteins involved in intracellular sorting, secretion and quality control

In eukaryotic systems, many components are targeted to different intracellular organelles, and the ability to alter localization and secretion is a valuable path for cell engineering [41]. For example, most of the proteins that are destined to the plasma membrane or the extracellular environment are first targeted to the endoplasmic reticulum (ER): there, the proteins are either inserted into the ER membrane or translocated into the ER lumen via the universally conserved Sec translocon, and packed into vesicles and trafficked to the plasma membrane via the Golgi network [14]. Although it is known that the gut fungi secrete a large number of biomass degrading enzymes, very little is known about the molecular details underlying protein trafficking in these understudied primitive eukaryotes.

As is shown in Fig. 3, we find that many gut fungal transcripts encode proteins that function in the biogenesis and intracellular trafficking of proteins; some of these components have previously been identified in *Orpinomyces* sp. [35]. For example, parts of the general secretory pathway (TCDB 3.A.5) are easily identified by homology, including four signal recognition particle (SRP) proteins, (SRP14, SRP54, SRP68 and SRP72); both SRP receptor subunits and a heterotrimeric Sec61 translocon as well as Sec62/63 [14]. Further, we find some 30 proteins that are homologous to heat shock proteins (TCDB 1.A.33), and proteins that belong to the endoplasmic reticular retrotranslocon family (TCDB 3.A.16) and that are implicated in protein folding and quality control [42]. We also find evidence for vesicular trafficking and membrane remodeling, in several Synaptosomal Vesicle Fusion Pore proteins (a.k.a. SNAREs) (TCDB 1.F.1); Synaptic Vesicle Associated Calcium Channels (1.A.55); and Annexin-like Proteins (TCDB 1.A.31) that are involved in the trafficking of vesicles and modulation of cell shape [43, 44].

Anaerobic gut fungi have intracellular hydrogenosomes that are related to the mitochondria of aerobic eukaryotes, which generate ATP by substrate-level phosphorylation [45, 46]. Apart from the above mentioned heat shock proteins, of which a subset may be located to the hydrogenosomes, we find evidence for components that are homologous to the mitochondrial and chloroplast import machinery (TCDB 1.B.33, 1.B.8, 3.A.8, 3.A.9), such as the central mitochondrial import receptor TOM40, the inner membrane translocases TIM22 and TIM23, and accessory factors TIM9 and TIM10 [47, 48]. Further, and although it is not entirely clear whether gut fungi have peroxisomes as such, we find evidence for the peroxisomal import machinery (TCDB 3.A.20); as well as many subunits of the large Nuclear Pore Complex and proteins that are implied in the maturation and nuclear export of RNA (TCDB 1.I.1, 3.A.18, 3.A.22, 9.A.50) [49, 50]. Finally, the ‘Other’ category captures proteins that are involved in energy conversion (TCDB 3.D.1 and 3.D.10), fatty acid translocators (TCDB 4.C.1), accessory factors (TCDB 8), and incompletely associated transport systems (TCDB 9).

### Potential transporters for biotechnology and strain engineering

**Fig. 4:**
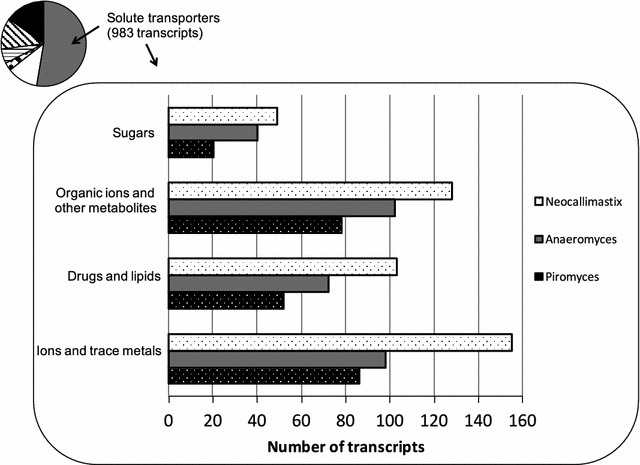
Substrates of 983 solute transporter components identified in three gut fungal strains, based on hits in TCDB. The proteins were sorted into these categories based on TCDB homology using a stringent 70% coverage criterion of both subject and query, with and E-value cutoff of 10^−3^. In the case of multiple matches, the match with lowest E-value was taken. Total number of transcripts encoding putative small-solute transporters in *Neocallimastix*: 435 transcripts; *Anaeromyces*: 312 transcripts; *Piromyces*: 236 transcripts

Virtually any solute in cells has to pass through a membrane-embedded transporter; this is true for ions and large molecules as well as for small molecules like water and glycerol [51, 52]. Given the ability of anaerobic fungi to persist in a competitive, lignocellulose rich environment, we hypothesize that their membrane proteome must therefore be well stocked with components that sense sugars and metabolites, selectively transport them, and extrude waste products or secondary metabolites. As shown in Fig. 4, in all three fungal strains we find a number of putative transporters for sugars and metabolites such as amino acids, organic ions, and nucleotides; putative drug transporters and lipid flippases; and channels and pumps for ions and trace metals.

#### Transporters for sugars, organic ions and other metabolites

417 transcripts in the three gut fungal strains encode transport components that are involved in the uptake or extrusion of sugars and other organic metabolites, which are the end products of biomass breakdown (Fig. 4). Sugar transporters are attractive targets for microbial engineering, and several efforts have been made to identify and engineer transporters that enhance the uptake of underutilized sugars. For example, transporters that mediate flux of five-carbon sugars derived from hemicellulose could open the way for pentose sugar metabolism in yeasts [5, 53–56]. Eukaryotic sugar uptake systems typically belong to the major facilitator superfamily (MFS) (TCDB 2.A.1); the solute sodium symporter family (SSS) (TCDB 2.A.2); and the recently characterized Sugars Will Eventually be Exported Transporter family (SWEET) (TCDB 2.A.123) [57]. These proteins are mostly secondary carriers, and although some function as uniporters, most couple the transport of the solute to the downhill transport of ions such as protons or sodium. As shown in Table 1, all these families are represented in the three fungi: in total we find 24 MFS transporters; 7 SSS transporters and 10 SWEET transporters. Using the fifth digit of the TCDB system we can tentatively assign substrates to a few of the proteins: mannose, fructose, xylose, sucrose, cellobiose and myoinositol, however without experimental characterization, these homology-based assignments remain putative [58].

**Table 1:** Putative sugar uptake systems identified in three gut fungal strains

	Neocallimastix	Anaeromyces	Piromyces
MFS	12	5	7
SSS	2	4	1
SWEET	5	3	2
ABC	30	28	10
Total	49	40	20

**Fig. 5:**
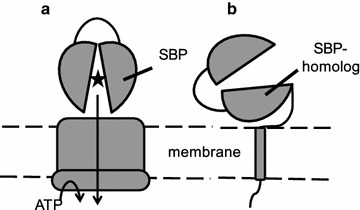
Prokaryotic SBPs and gut fungal SBP-homologs. **a** Shows a cartoon of a prokaryotic ABC-importer. The SBP delivers the substrate to the membrane embedded component that utilizes ATP to translocate the substrate across the membrane. **b** Shows a cartoon of gut fungal SBP-homolog identified from transcriptomics with currently unknown function. The identified gut fungal SBPs are homologous to Type II SBPs and have one or more predicted amino- or carboxy-terminal transmembrane helix with no known homology to other proteins

Unexpectedly, 60% of the predicted sugar transporter components that we have identified in the three fungi are homologous to the substrate binding protein (SBP) of prokaryotic solute uptake systems that belong to the ATP binding cassette (ABC) transport superfamily (TCDB 3.A.1). Although ABC transporters as such are abundant in all kingdoms of life, SBP-coupled ABC uptake-systems have to date only been found in prokaryotes [59, 60]. Typically, these modular transport systems consist of two cytoplasmic nucleotide-binding domains, two transmembrane domains, and an extracellular SBP encoded on up to four different polypeptides [30, 60] (Fig. 5a). The extracytoplasmic SBP delivers the substrate to the membrane embedded domain that utilizes ATP to pump the substrate across the membrane [61]. Based on structural details, SBPs and SBP-domains can be divided into three classes: Type I (SCOP superfamily SSF53822), Type II (SCOP superfamily SSF53850), and Type III (SCOP superfamily SSF53807) [62–65]. While SBP-coupled ABC uptake systems seem to be exclusively prokaryotic, SBP-domains are found in eukaryotic membrane proteins such as guanylyl cyclase-linked natriuretic peptide receptors, ligand-gated ion channels and class C GPCRs [62, 63, 66, 67]. The eukaryotic membrane bound SBP-domains are typically Type I, with the exception of ligand-gated ion-channels that have a Type II domain encoded by two non-consecutive parts of the polypeptide chain [68]. Strikingly however, the SBP proteins that we find in the gut fungi are invariably similar to Type II proteins, and while some of them are predicted to have transmembrane helices, there is nothing in the sequence that immediately suggests that they form e.g. a ligand gated-ion channel (Fig. 5b). Based on similarity to proteins in the TCDB, the gut fungal SBPs are related to palatinose, trehalose/maltose/sucrose and xylobiose-binding proteins from the bacteria *Agrobacterium tumefaciens, Erwinia rhapontici, Sinorhizobium melioti, Streptomyces coelicolor, Streptomyces thermoviolaceus, Thermus thermophilus, Thermotogae, Rhodobacter sphaeroides,* and the archaeon *Thermococcus litoralis*. Most of these microorganisms are associated with soil and plants and it is not unlikely that the fungi have acquired the genes by horizontal gene transfer [19].

Although we failed to identify any other putative ABC-importer components among the fungal transcripts, i.e. the membrane-embedded and cytoplasmic nucleotide binding domains, it is possible that these remain to be identified in the genomes. Alternatively, the sequence similarity to other transporters may be so low that our stringent 70% criterion fails to identify the other ABC transporter components. In any case, the isolated SBP proteins are not likely to function as transmembrane carriers on their own; however, it is possible that some of these have functions that we cannot easily discern from primary sequence alone. It is tempting to speculate as to their function in the fungi: do these SBP proteins communicate with fungal transporters, or do they act as sugar sequesters that grasp onto the sugars that the extracellular cellulolytic machinery produces? This could conceivably increase the local sugar concentration around the fungus and lead to increased sugar uptake. Further, SBP proteins in prokaryotes are known to communicate with chemotaxis proteins, and it is possible that the gut fungal SBPs play a role in directing the fungal zoospores to nutrient sources by a yet unknown mechanism [69].

In addition to sugar transporters, we find a diverse repertoire of transporters for several classes of organic ions and amino acids (TCDB 2.A.1.19, 2.A.18, 2.A.22, 2.A.79, 2.A.85), ammonia (TCDB 1.A.11) and sugar alcohols such as glycerol (TCDB 2.A.50). There are also putative channels for formate and nitrate (TCDB 1.A.16), and transporters for nucleotides and nucleosides (TCDB 2.A.7.11, 2.A.7.12). It is worth to note that each fungal strain has more than ten transcripts encoding proteins that are homologous to mitochondrial carriers (TCDB 2.A.29). These proteins are typically involved in the compartmental exchange of solutes such as ATP/ADP, and are likely localized to the hydrogenosomes [70–72].

#### Promiscuous drug extruders and lipid flippases

227 transcripts in the three gut fungal strains encode putative promiscuous drug extruders and lipid flippases (Fig. 4), which could enhance the tolerance and yields of metabolically engineered chemical production strains [4, 8, 73, 74]. In all three fungal strains, we find a number of Drug:Proton antiporter proteins (DHA) (TCDB 2.A.1.2, 2.A.1.3; 55 proteins in total). DHA proteins belong to the MFS and are abundant in the fungal kingdom and believed to be involved in the extrusion of various mycotoxins such as polyketides [75, 76]. Although implicated in chemical stress tolerance, drug resistance and pathogenicity, DHA transporters are abundant also in non-pathogenic fungi and thus their role is not entirely clear, however it has been speculated that some of the extruded compounds are used to restrain microbial competition [77, 78]. In addition, we find evidence for a number of transporters from the multi antimicrobial extrusion (MATE) family (TCDB 2.A.66, 19 proteins in total) and a number of ABC exporters that are involved in broad specificity drug resistance (TCDB 3.A.1.201, 24 proteins in total).

Lipid flippases are involved in the organization of lipids within cellular membranes, the modulation of the fluidity of cell membranes, and the formation of extracellular glycoconjugates and polysaccharides [79, 80]. In each fungal strain, we find evidence for lipid flippases, primarily from the ABC transporter family ABCA (3.A.1.211), and the P-type ATPase superfamily (TCDB 3.A.3.8). As the substrates of lipid flippases are hydrophobic and oil-like, they could conceivably be engineered for biofuel tolerance or the production of e.g. high-value terpenoid compounds [81, 82].

#### Transporters for inorganic ions and trace metals

339 transcripts in the three gut fungal strains encode channels and pumps for inorganic ions and trace metals (Fig. 4). Inorganic ion transporters are typically involved in the maintenance of cellular pH homeostasis, signal transduction, and the buildup of ion gradients that the cell uses for downstream applications [83]. Alkali and transition metals are important enzymatic and structural cofactors in a wide range of enzymes. These transporters may thus enhance the stability and enzymatic performance of microbial production strains; in addition, metal transporters can be used for the detection and bioremediation of heavy-metal contaminations [84–86]. Apart from voltage-gated potassium channels and chloride channels (TCDB 1.A.1, 2.A.40), we find several subunits of V-type ATPases and P-type ATPases that are typically involved in the pumping of protons and other cations across cellular membranes, although some P-type ATPases have also been implied in lipid transport (TCDB 3.A.2, TCDB 3.A.3) [79, 80]. There are a handful of proteins that are similar to bacterial arsenite transporters (TCDB 2.A.59), as well as putative transporters for zinc, iron and magnesium (TCDB 1.A.26, 2.A.5, 2.A.89).

### Anaerobic gut fungi possess novel GPCRs

Next, we sought to investigate unique receptors identified from sequencing all three strains of gut fungi, which may have a role in sugar sensing. Across genera, we identified a wealth of GPCRs, which is the largest receptor class in eukaryotes [87]. Using the InterProScan tool and BLAST annotations, we identified 53 putative GPCRs in *N. californiae*, 25 GPCRs in *A. robustus* and 34 GPCRs in *P. finnis* (Table 2). The heptahelical GPCRs typically display an amino-terminal ligand-binding domain at the surface of the cell, recognize a wide range of ligands, and are involved in numerous sensory processes, cellular growth and development. Based on sequence analyses and phylogeny, GPCRs can be sorted into at least five (Glutamate, Rhodopsin, Adhesion, Frizzled and Secretin) or six (A-F) classes [88, 89]. Using the InterProScan tool, we determined that a small number of the GPCRs in these gut fungi are rhodopsin-like or possibly related to the cAMP receptors that were first identified in the slime mold *Dictyostelium discoideum* (Dicty-CAR; IPR017452; IPR017981) (Table 2) [90]. Interestingly, the Dicty-CAR receptors are implicated in cell differentiation in *D. discoidum*, and it is possible that these GPCRs are involved in the complex gut fungal life cycle, which involves a motile zoospore state and a sessile state that burrows into plant material [90].

**Table 2:** Putative GPCRs in the three gut fungal strains

	Neocallimastix	Anaeromyces	Piromyces
Rhodopsin/dicty-CAR	2	1	2
Class C (glutamate)	51	24	32
Total	53	25	34

The vast majority of the gut fungal GPCRs that we identified in this study have the highest similarity to Glutamate, or class C GPCRs (a.k.a. class 3; IPR017978), a class that until recently was believed to be absent from the fungal kingdom (Table 2) [91]. Class C GPCRs comprise metabotropic glutamate receptors, calcium sensing receptors, sweet taste receptors and gamma aminobutyric acid receptors type B (GABA_B_) [92]. These receptors typically have a long (>400 amino acids) ligand-binding domain called the Atrial Natriuretic Factor receptor (ANF) domain (IPR001828), which is related to prokaryotic amino acid binding proteins that belong to the structural SBP Type I superfamily (SCOP superfamily SSF53822) [66, 67]. With the exception of GABA_B_ receptors, all known class C GPCRs also have a pattern of 9 conserved Cysteine residues between the amino-terminal domain and the seven transmembrane helices (IPR011500) [93].

#### Gut fungal class C GPCRs have a non-canonical architecture with putative carbohydrate-binding domains

**Fig. 6:**
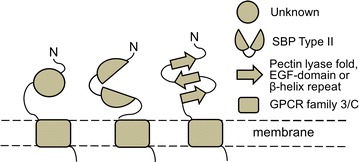
Domain architecture of gut fungal class C GPCRs identified from transcriptome data. All class C GPCRs are predicted to have a long amino-terminal domain and seven carboxy-terminal transmembrane helices. The amino-terminal domain ranges from 200 to 1600 amino acid residues with the average length being 600 residues. Around 30% of the putative GPCRs are predicted to have an extracellular pectin lyase fold (IPR011050; IPR012334), parallel beta-helix repeats (IPR006626), and/or an EGF-like domain (IPR000742). Around 50% of the GPCRs are predicted to have a domain that is homologous to SBP Type II (a.k.a. Periplasmic binding protein-like II, SCOP superfamily SSF53850). Several putative GPCRs do not have any apparent homology to known InterPro domains. In approximately 30% of the cases we can identify a canonical ER targeting signal peptide at the very aminoterminus (not shown). *N* amino-terminus. For more details, see Additional file 2: Figure S2

As shown in Fig. 6, all gut fungal class C GPCRs identified in this study are predicted to have the characteristic large extracellular domain, sometimes reaching well over 1000 amino acid residues (Fig. 6; Additional file 2: Figure S2). However, instead of an ANF domain, around 30% of the GPCRs display a pectin lyase fold/virulence factor (IPR011050; IPR012334), sometimes accompanied by several parallel beta-helix repeats (IPR006626) and in a few cases by an EGF-like domain (IPR000742) (Fig. 6; Additional file 2: Figure S2). Pectin is a major component of plant cell walls, and pectin and pectate lyases are virulence factors that are secreted by plant pathogens [94, 95]. Both enzymes display beta strand repeats, a common motif among enzymes that recognize carbohydrate substrates [96]. EGF-domains are typically around 40 amino acid residues long and found in many different proteins in one or multiple copies [97]. EGF domains contain a motif of six cysteines; and some EGF domains are known to bind calcium. Notably, EGF-domains are found at the amino-terminus of so called Adhesion GPCRs (Class2/B), that are characterized by very long extracellular domains with multiple functional domains; however nothing in the sequences identified here suggest that the gut fungal GPCRs belong to class B [98].

Interestingly, almost half of the gut fungal GPCRs have an amino-terminal SBP Type II-domain (SCOP superfamily SSF53850). As mentioned previously, this domain is related to—yet structurally different from—the ANF domain that is found in metazoan class C GPCRs. In the gut fungal GPCRs, the Type II domain is invariably similar to prokaryotic substrate binding proteins that are associated with sugar uptake systems (Fig. 5). In agreement with our findings, it was recently shown that fungal class C GPCRs display an unprecedented variety of amino-terminal domains, among them SBP Type II domains that resemble the domains that are identified in this study [91]. Strikingly however, we failed to find a single example of a class C GPCR with the ANF domain, which is the dominating amino-terminal domain in all characterized class C GPCRs. Also, although several of the gut fungal GPCRs have up to 10 cysteines in their amino-terminal domain, the sequences do not align to the conserved nine cysteines domain. It has been suggested that class C GPCRs evolved through the fusion of a prokaryotic SBP and a bacteriorhodopsin [67, 92, 99–101]. The diversity of amino-terminal domains in our gut fungal GPCRs corroborate that these fusions may have happened more than once and between different genes.

## Conclusions

Integral membrane proteins are a vital component of all living cells, and it is becoming increasingly clear that membrane-embedded transporters and receptors are essential for the engineering and stability of microbial production strains. Here, we searched for integral membrane proteins in transcriptomic data collected from three different genera of lignocellulolytic anaerobic gut fungi that are highly relevant for applications that convert renewable biomass into value-added compounds. We hypothesized that these extraordinarily persistent microorganisms possess a wide variety of solute transporters and receptors that are involved in the uptake and recognition of carbohydrates.

A relatively simple strategy that integrates transcriptomics with sequence similarity-based comparisons revealed a treasure trove of novel membrane proteins from anaerobic fungi that are of broad biotechnological interest. In the absence of high-quality genomic information, the resolution of the transcriptome is indeed remarkable, capturing the “active” part of the genome most critical to the lifestyle of these fungi. Here, we identified hundreds of novel sugar transporters and solute extruders from these unexplored fungi, which can be used to bolster substrate acquisition and tolerance in model microbes like *Escherichia coli*, *Saccharomyces cerevisiae*, and even more evolved fungi. Additionally, we find transcripts that encode universally conserved proteins, *e.g.* all three subunits of the heterotrimeric Sec61 translocon as well as other conserved components of the general secretory pathway that provide a path forward for understanding and engineering protein secretion in these early-branching fungi.

Of particular interest for future characterization are the unique and seemingly prokaryotic transporters and receptors identified here that bear unexpected N-terminal domains with putative sugar binding and transport functionalities. Along with transcripts that encode membrane-anchored carbohydrate-binding domains, we speculate that these domains may be involved in carbohydrate sensing and sequestration that convey a competitive edge to these slow growing fungi in microbial communities. Overall, this study reveals entirely new subsets of membrane protein transporters and receptors from nature to enhance biomass breakdown and substrate utilization.

## Methods

### Fungal strains and RNA isolation

Three novel gut fungal species from distinct genera of Neocallimastigomycota (*Piromyces finnis, Anaeromyces robustus,* and *Neocallimastix californiae*) were isolated from environmental samples [26] for study and analysis. We grew these cultures in 10 mL batch cultures of anaerobic Medium C, on a range of diverse fibrous and soluble carbon substrates (e.g. reed canary grass, glucose, cellobiose) before extracting their total RNA content with the RNeasy^®^ Mini Kit (Qiagen, Valencia, CA) as previously described [19]. To maximize the number of transcripts observed, we pooled RNA preps from different substrates in equimolar proportions, as measured by a NanoDrop 2000 (ThermoScientific, Wilmington, DE), before sequencing.

### Transcriptome acquisition and annotation

Fungal transcriptomes were previously acquired for all fungal strains, which serve as the base dataset for this study [19]. Briefly, RNA sample integrity was validated with a 2200 Tapestation (Agilent Technologies, Santa Clara, CA). Intact samples were used to generate strand-specific cDNA libraries, sequenced on an Illumina HiSeq (Illumina, San Diego, CA) and annotated as described previously to obtain *de novo* transcriptomes [19]. Briefly, we annotated the transcriptomes using the automated BLAST2GO pipeline [16], which analyzes sequences for similarity (*blastx*) and protein domains via hidden Markov model signatures (InterProScan). Significant hits had an E value of ≤10^−3^. Annotations from this pipeline included a protein description, delineation of internal domains, functions described by Gene Ontology (GO) terms associated with these domains, and assignment of Enzyme Comission (EC) numbers, if available. We were also able to identify non-coding antisense transcripts within the strand-specific transcriptome on the basis of the annotation reading frame (−1, −2, −3).

### Identification of integral membrane and other secreted proteins

We identified secreted proteins within the transcriptomes by parsing the annotation files provided by BLAST2GO for InterPro domain hits. Transmembrane domains were predicted by Phobius [102] and TMHMM [13], and signal peptides were predicted by Phobius and SignalP [15].

### Filtering and classifying the transcriptome

Membrane protein candidates were classified into one of four primary roles on the basis of their associated GO Terms in the precedence order: ‘Transport’, ‘Sensing and Signaling’, ‘Catalysis’, ‘Other’, and ‘Unknown’. Each GO annotation was parsed and searched for functional keywords as follows: *Transport* encompasses all membrane proteins with a stated “transport”, “symport”, or “V-type ATPase” role such as ABC transporters, P-type ATPase ion pumps, solute symporters, antiporters, and uniporters; *Sensing and Signaling* includes proteins annotated with a “receptor”, “signal”, or “sensor” function; *Catalysis* proteins all have roles that terminate in ‘-ase’; *Unknown* includes proteins that cannot be assigned a GO term while *Other* counts the remaining unassigned proteins. To better represent the total protein count encoded in the transcriptome, proteins with multiple functions are only assigned to the role of highest precedence. For example, ABC transporters with both transport and catalytic ATPase functions are binned only once under *Transport*.

### Transporter analysis

The translated amino acid sequence for each transcript was aligned to the transporter classification system database (TCDB) [17] using a local installation of NCBI BLAST’s *blastp*. TCDB database was downloaded January 15, 2015. To increase the confidence in our predictions, we filtered the results to include only hits that covered at least 70% of the amino acid sequences of both the query and the subject. After filtering by coverage, the hit with smallest E-value was selected, with a maximum cutoff of 10^−3^.

### Identification of putative GPCRs

Transcripts with putative GPCR function were identified by searching the functional annotations provided by NCBI BLAST and InterPro databases for keywords ‘GPCR’ and ‘G-protein coupled receptor’. From this subset, only sequences that contained between 7 and 9 transmembrane domains as identified by transmembrane hidden markov models (TMHMM). This ensured that transcripts identified were full length GPCRs with 7 transmembrane domains and allowed for the presence of hydrophobic signal sequences that may also be identified as transmembrane domains. Predicted N-terminal domains were identified by the InterPro based annotations present in the extracellular N-terminal region. These were identified by selecting all domains from the GPCRs that were present before the first of the seven transmembrane sequences typical of GPCRs, restricting the search to only the N-terminal extracellular region.

## Additional files

**Additional file 1: Figure S1.** Putative functions of fungal transporters based on transporter classification data base (TCDB) analysis, showing the mode of transport. 1868 fungal transporter components from three gut fungal strains were sorted based on TCDB homology using a stringent 70% coverage criterion. The mode of transport is indicated. Total number of transcripts encoding putative transporter components in *Neocallimastix*: 826 transcripts; *Anaeromyces*: 554 transcripts; *Piromyces*: 488 transcripts.

**Additional file 2: Figure S2.** Detailed domain architecture of gut fungal class C GPCRs as determined using the InterProScan tool. All class C GPCRs are predicted to have a long amino-terminal domain and seven carboxy-terminal transmembrane helices. The amino-terminal domain shows great variability. The predicted positions of the domains on the polypeptide chains are indicated.

**Additional file 3: Database 1.** The assembled transcriptome for *Neocallimastix* californiae.

**Additional file 4: Database 2.** The assembled transcriptome for *Anaeromyces* robustus.

**Additional file 5: Database 3.** The assembled transcriptome for *Piromyces* finnis.

**Additional file 6: Table S3.** Annotation of the *Piromyces finnis* transcriptome.

**Additional file 7: Table S1.** The transporter classification database (TCDB) hits identified in this study.

**Additional file 8: Table S2.** The G-protein coupled sequences (GPCRs) identified in this study.

## References

[1] Kell DB, Swainston N, Pir P, Oliver SG (2015). Membrane transporter engineering in industrial biotechnology and whole cell biocatalysis. Trends Biotechnol.

[2] Boyarskiy S, Tullman-Ercek D (2015). Getting pumped: membrane efflux transporters for enhanced biomolecule production. Curr Opin Chem Biol.

[3] Nieves LM, Panyon LA, Wang X (2015). Engineering sugar utilization and microbial tolerance toward lignocellulose conversion. Front Bioeng Biotechnol.

[4] Turner WJ, Dunlop MJ (2015). Trade-offs in improving biofuel tolerance using combinations of efflux pumps. ACS Synth Biol.

[5] Hector RE, Qureshi N, Hughes SR, Cotta MA (2008). Expression of a heterologous xylose transporter in a *Saccharomyces cerevisiae* strain engineered to utilize xylose improves aerobic xylose consumption. Appl Microbiol Biotechnol.

[6] Young E, Poucher A, Comer A, Bailey A, Alper H (2011). Functional survey for heterologous sugar transport proteins, using *Saccharomyces cerevisiae* as a host. Appl Environ Microbiol.

[7] Ha SJ, Galazka JM, Joong OhE, Kordić V, Kim H, Jin YS (2013). Energetic benefits and rapid cellobiose fermentation by *Saccharomyces cerevisiae* expressing cellobiose phosphorylase and mutant cellodextrin transporters. Metab Eng.

[8] Dunlop MJ, Dossani ZY, Szmidt HL, Chu HC, Lee TS, Keasling JD (2011). Engineering microbial biofuel tolerance and export using efflux pumps. Mol Syst Biol.

[9] Geddes RD, Wang X, Yomano LP, Miller EN, Zheng H, Shanmugam KT (2014). Polyamine transporters and polyamines increase furfural tolerance during xylose fermentation with ethanologenic *Escherichia coli* strain LY180. Appl Environ Microbiol.

[10] Yu AQ, Pratomo Juwono NK, Foo JL, Leong SSJ, Chang MW (2016). Metabolic engineering of *Saccharomyces cerevisiae* for the overproduction of short branched-chain fatty acids. Metab Eng.

[11] Benson DA, Cavanaugh M, Clark K, Karsch-Mizrachi I, Lipman DJ, Ostell J (2013). GenBank. Nucleic Acids Res.

[12] von Heijne G, Gavel Y (1988). Topogenic signals in integral membrane proteins. Eur J Biochem.

[13] Krogh A, Larsson B, von Heijne G, Sonnhammer EL (2001). Predicting transmembrane protein topology with a hidden Markov model: application to complete genomes. J Mol Biol.

[14] Rapoport TA (2007). Protein translocation across the eukaryotic endoplasmic reticulum and bacterial plasma membranes. Nature.

[15] Petersen TN, Brunak S, von Heijne G, Nielsen H (2011). SignalP 4.0: discriminating signal peptides from transmembrane regions. Nat Methods.

[16] Götz S, García-Gómez JM, Terol J, Williams TD, Nagaraj SH, Nueda MJ (2008). High-throughput functional annotation and data mining with the Blast2GO suite. Nucleic Acids Res.

[17] Saier MH, Reddy VS, Tamang DG, Västermark A (2014). The transporter classification database. Nucleic Acids Res.

[18] Orpin CG (1975). Studies on the rumen flagellate Neocallimastix frontalis. J Gen Microbiol.

[19] Solomon KV, Haitjema CH, Henske JK, Gilmore SP, Borges-Rivera D, Lipzen A (2016). Early-branching gut fungi possess a large, comprehensive array of biomass-degrading enzymes. Science.

[20] Theodorou MK, Mennim G, Davies DR, Zhu WY, Trinci AP, Brookman JL (1996). Anaerobic fungi in the digestive tract of mammalian herbivores and their potential for exploitation. Proc Nutr Soc.

[21] Wood TM, Wilson CA (1995). Studies on the capacity of the cellulase of the anaerobic rumen fungus Piromonas communis P to degrade hydrogen bond-ordered cellulose. Appl Microbiol Biotechnol.

[22] Gruninger RJ, Puniya AK, Callaghan TM, Edwards JE, Youssef N, Dagar SS (2014). Anaerobic fungi (phylum Neocallimastigomycota): advances in understanding their taxonomy, life cycle, ecology, role and biotechnological potential. FEMS Microbiol Ecol.

[23] Haitjema CH, Solomon KV, Henske JK, Theodorou MK, O’Malley MA (2014). Anaerobic gut fungi: advances in isolation, culture, and cellulolytic enzyme discovery for biofuel production. Biotechnol Bioeng.

[24] Krause DO, Nagaraja TG, Wright ADG, Callaway TR (2013). Board-invited review: rumen microbiology: leading the way in microbial ecology. J Anim Sci.

[25] Chaucheyras-Durand F, Ossa F (2014). REVIEW: the rumen microbiome: Composition, abundance, diversity, and new investigative tools. Prof Anim Sci.

[26] Li GJ, Hyde KD, Zhao RL, Hongsanan S, Abdel-Aziz FA, Abdel-Wahab MA (2016). Fungal diversity notes 253–366: taxonomic and phylogenetic contributions to fungal taxa. Fungal Divers..

[27] Sonnhammer EL, von Heijne G, Krogh A (1998). A hidden Markov model for predicting transmembrane helices in protein sequences. Proc Int Conf Intell Syst Mol Biol.

[28] Teunissen MJ, Op den Camp HJM (1993). Anaerobic fungi and their cellulolytic and xylanolytic enzymes. Antonie Van Leeuwenhoek.

[29] Ashburner M, Ball CA, Blake JA, Botstein D, Butler H, Cherry JM (2000). Gene ontology: tool for the unification of biology. The Gene Ontology Consortium. Nat Genet.

[30] Theodoulou FL, Kerr ID (2015). ABC transporter research: going strong 40 years on. Biochem Soc Trans.

[31] Lemmon MA, Schlessinger J (2010). Cell signaling by receptor tyrosine kinases. Cell.

[32] Hubert P, Sawma P, Duneau J-P, Khao J, Hénin J, Bagnard D (2014). Single-spanning transmembrane domains in cell growth and cell-cell interactions. Cell Adh Migr.

[33] Kemp G, Cymer F (2014). Small membrane proteins - elucidating the function of the needle in the haystack. Biol Chem.

[34] Zviling M, Kochva U, Arkin IT (2007). How important are transmembrane helices of bitopic membrane proteins?. Biochim Biophys Acta.

[35] Youssef NH, Couger MB, Struchtemeyer CG, Liggenstoffer AS, Prade RA, Najar FZ (2013). The genome of the anaerobic fungus Orpinomyces sp. strain C1A reveals the unique evolutionary history of a remarkable plant biomass degrader. Appl Environ Microbiol.

[36] Altschul SF, Gish W, Miller W, Myers EW, Lipman DJ (1990). Basic local alignment search tool. J Mol Biol.

[37] Almén MS, Nordström KJ, Fredriksson R, Schiöth HB (2009). Mapping the human membrane proteome: a majority of the human membrane proteins can be classified according to function and evolutionary origin. BMC Biol.

[38] Wimley WC (2002). Toward genomic identification of beta-barrel membrane proteins: composition and architecture of known structures. Protein Sci.

[39] Bigelow HR, Petrey DS, Liu J, Przybylski D, Rost B (2004). Predicting transmembrane beta-barrels in proteomes. Nucleic Acids Res.

[40] Beck M, Förster F, Ecke M, Plitzko JM, Melchior F, Gerisch G (2004). Nuclear pore complex structure and dynamics revealed by cryoelectron tomography. Science.

[41] Blobel G (1980). Intracellular protein topogenesis. Proc Natl Acad Sci USA.

[42] Amm I, Sommer T, Wolf DH (2014). Protein quality control and elimination of protein waste: the role of the ubiquitin-proteasome system. Biochim Biophys Acta.

[43] Wickner W, Schekman R (2008). Membrane fusion. Nat Struct Mol Biol.

[44] Gerke V, Creutz CE, Moss SE (2005). Annexins: linking Ca2+ signalling to membrane dynamics. Nat Rev Mol Cell Biol.

[45] Marvin-Sikkema FD, Pedro Gomes TM, Gottschal JC, Prins RA, Marvin-Sikkema FD (1993). Characterization of hydrogenosomes and their role in glucose metabolism of Neocallimastix sp. L2. Arch Microbiol.

[46] Makiuchi T, Nozaki T (2014). Highly divergent mitochondrion-related organelles in anaerobic parasitic protozoa. Biochimie.

[47] Becker T, Gebert M, Pfanner N, van der Laan M (2009). Biogenesis of mitochondrial membrane proteins. Curr Opin Cell Biol.

[48] Strittmatter P, Soll J, Bölter B (2010). The chloroplast protein import machinery: a review. Methods Mol Biol.

[49] Platta HW, Hagen S, Erdmann R (2013). The exportomer: the peroxisomal receptor export machinery. Cell Mol Life Sci.

[50] Dickmanns A, Kehlenbach RH, Fahrenkrog B (2015). Nuclear pore complexes and nucleocytoplasmic transport: from structure to function to disease. Int Rev Cell Mol Biol.

[51] Preston GM, Carroll TP, Guggino WB, Agre P (1992). Appearance of water channels in Xenopus oocytes expressing red cell CHIP28 protein. Science.

[52] Kaldenhoff R, Kai L, Uehlein N (2014). Aquaporins and membrane diffusion of CO_2_ in living organisms. Biochim Biophys Acta.

[53] Young EM, Comer AD, Huang H, Alper HS (2012). A molecular transporter engineering approach to improving xylose catabolism in *Saccharomyces cerevisiae*. Metab Eng.

[54] Young EM, Tong A, Bui H, Spofford C, Alper HS (2014). Rewiring yeast sugar transporter preference through modifying a conserved protein motif. Proc Natl Acad Sci USA.

[55] Farwick A, Bruder S, Schadeweg V, Oreb M, Boles E (2014). Engineering of yeast hexose transporters to transport d-xylose without inhibition by d-glucose. Proc Natl Acad Sci USA.

[56] Wang M, Yu C, Zhao H (2015). Directed evolution of xylose specific transporters to facilitate glucose-xylose co-utilization. Biotechnol Bioeng.

[57] Chen LQ, Cheung LS, Feng L, Tanner W, Frommer WB (2015). Transport of sugars. Annu Rev Biochem.

[58] Mishra NK, Chang J, Zhao PX, Fotiadis D (2014). Prediction of membrane transport proteins and their substrate specificities using primary sequence information. PLoS ONE.

[59] Rees DC, Johnson E, Lewinson O (2009). ABC transporters: the power to change. Nat Rev Mol Cell Biol.

[60] ter Beek J, Guskov A, Slotboom DJ (2014). Structural diversity of ABC transporters. J Gen Physiol.

[61] van der Heide T, Poolman B (2002). ABC transporters: one, two or four extracytoplasmic substrate-binding sites?. EMBO Rep.

[62] Fukami-Kobayashi K, Tateno Y, Nishikawa K (1999). Domain dislocation : a change of core structure in periplasmic binding proteins in their evolutionary history. J Mol Biol.

[63] Berntsson RP, Smits SHJ, Schmitt L, Slotboom DJ, Poolman B (2010). A structural classification of substrate-binding proteins. FEBS Lett.

[64] Gough J, Karplus K, Hughey R, Chothia C (2001). Assignment of homology to genome sequences using a library of hidden Markov models that represent all proteins of known structure. J Mol Biol.

[65] Murzin AG, Brenner SE, Hubbard T, Chothia C (1995). SCOP: a structural classification of proteins database for the investigation of sequences and structures. J Mol Biol.

[66] O’Hara PJ, Sheppard PO, Thøgersen H, Venezia D, Haldeman BA, McGrane V (1993). The ligand-binding domain in metabotropic glutamate receptors is related to bacterial periplasmic binding proteins. Neuron.

[67] Felder CB, Graul RC, Lee AY, Merkle HP, Sadee W (1999). The Venus flytrap of periplasmic binding proteins: an ancient protein module present in multiple drug receptors. AAPS PharmSci.

[68] Armstrong N, Sun Y, Chen GQ, Gouaux E (1998). Structure of a glutamate-receptor ligand-binding core in complex with kainate. Nature.

[69] Spurlino JC, Lu GY, Quiocho FA (1991). The 2.3-A resolution structure of the maltose- or maltodextrin-binding protein, a primary receptor of bacterial active transport and chemotaxis. J Biol Chem.

[70] van der Giezen M, Slotboom DJ, Horner DS, Dyal PL, Harding M, Xue GP (2002). Conserved properties of hydrogenosomal and mitochondrial ADP/ATP carriers: a common origin for both organelles. EMBO J.

[71] Voncken F, Boxma B, Tjaden J, Akhmanova A, Huynen M, Verbeek F (2002). Multiple origins of hydrogenosomes: functional and phylogenetic evidence from the ADP/ATP carrier of the anaerobic chytrid Neocallimastix sp.. Mol Microbiol.

[72] Haferkamp I, Hackstein JHP, Voncken FGJ, Schmit G, Tjaden J (2002). Functional integration of mitochondrial and hydrogenosomal ADP/ATP carriers in the *Escherichia coli* membrane reveals different biochemical characteristics for plants, mammals and anaerobic chytrids. Eur J Biochem.

[73] Chen B, Ling H, Chang MW (2013). Transporter engineering for improved tolerance against alkane biofuels in *Saccharomyces cerevisiae*. Biotechnol Biofuels.

[74] Frederix M, Hütter K, Leu J, Batth TS, Turner WJ, Rüegg TL (2014). Development of a native *Escherichia coli* induction system for ionic liquid tolerance. PLoS ONE.

[75] Coleman JJ, Mylonakis E (2009). Efflux in fungi: la pièce de résistance. PLoS Pathog.

[76] Sá-Correia I, dos Santos SC, Teixeira MC, Cabrito TR, Mira NP (2009). Drug:H+ antiporters in chemical stress response in yeast. Trends Microbiol.

[77] Xu X, Chen J, Xu H, Li D (2014). Role of a major facilitator superfamily transporter in adaptation capacity of Penicillium funiculosum under extreme acidic stress. Fungal Genet Biol.

[78] Costa C, Dias PJ, Sá-Correia I, Teixeira MC (2014). MFS multidrug transporters in pathogenic fungi: do they have real clinical impact?. Front Physiol.

[79] Pomorski T, Menon AK (2006). Lipid flippases and their biological functions. Cell Mol Life Sci.

[80] Montigny C, Lyons J, Champeil P, Nissen P, Lenoir G (2015). On the molecular mechanism of flippase- and scramblase-mediated phospholipid transport. Biochim Biophys Acta.

[81] Gomès E, Jakobsen MK, Axelsen KB, Geisler M, Palmgren MG (2000). Chilling tolerance in Arabidopsis involves ALA1, a member of a new family of putative aminophospholipid translocases. Plant Cell.

[82] Rodríguez-Vargas S, Sánchez-García A, Martínez-Rivas JM, Prieto JA, Randez-Gil F (2007). Fluidization of membrane lipids enhances the tolerance of *Saccharomyces cerevisiae* to freezing and salt stress. Appl Environ Microbiol.

[83] Cyert MS, Philpott CC (2013). Regulation of cation balance in *Saccharomyces cerevisiae*. Genetics.

[84] Wang J, Zhang B, Zhang J, Wang H, Zhao M, Wang N (2014). Enhanced succinic acid production and magnesium utilization by overexpression of magnesium transporter mgtA in *Escherichia coli* mutant. Bioresour Technol.

[85] Duprey A, Chansavang V, Frémion F, Gonthier C, Louis Y, Lejeune P (2014). “NiCo Buster”: engineering *E. coli* for fast and efficient capture of cobalt and nickel. J Biol Eng.

[86] Kim SK, Lee BS, Wilson DB, Kim EK (2005). Selective cadmium accumulation using recombinant *Escherichia coli*. J Biosci Bioeng.

[87] Lagerström MC, Schiöth HB (2008). Structural diversity of G protein-coupled receptors and significance for drug discovery. Nat Rev Drug Discov.

[88] Fredriksson R, Lagerström MC, Lundin LG, Schiöth HB (2003). The G-protein-coupled receptors in the human genome form five main families. Phylogenetic analysis, paralogon groups, and fingerprints. Mol Pharmacol.

[89] Kolakowski LF (1994). GCRDb: a G-protein-coupled receptor database. Receptors Channels.

[90] Louis JM, Ginsburg GT, Kimmel AR (1994). The cAMP receptor CAR4 regulates axial patterning and cellular differentiation during late development of Dictyostelium. Genes Dev.

[91] Krishnan A, Almén MS, Fredriksson R, Schiöth HB (2012). The origin of GPCRs: identification of mammalian like Rhodopsin, Adhesion, Glutamate and Frizzled GPCRs in fungi. PLoS ONE.

[92] Pin JP, Galvez T, Prézeau L (2003). Evolution, structure, and activation mechanism of family 3/C G-protein-coupled receptors. Pharmacol Ther.

[93] Liu X, He Q, Studholme DJ, Wu Q, Liang S, Yu L (2004). NCD3G: a novel nine-cysteine domain in family 3 GPCRs. Trends Biochem Sci.

[94] Marin-Rodriguez MC (2002). Pectate lyases, cell wall degradation and fruit softening. J Exp Bot.

[95] Mayans O, Scott M, Connerton I, Gravesen T, Benen J, Visser J (1997). Two crystal structures of pectin lyase A from Aspergillus reveal a pH driven conformational change and striking divergence in the substrate-binding clefts of pectin and pectate lyases. Structure.

[96] Jenkins J, Mayans O, Pickersgill R (1998). Structure and evolution of parallel beta-helix proteins. J Struct Biol.

[97] Davis CG (1990). The many faces of epidermal growth factor repeats. New Biol.

[98] Bjarnadóttir TK, Fredriksson R, Schiöth HB (2007). The adhesion GPCRs: a unique family of G protein-coupled receptors with important roles in both central and peripheral tissues. Cell Mol Life Sci.

[99] Zhang Z (2012). A brief review on the evolution of GPCR: conservation and diversification. Open J Genet.

[100] Zhang Z, Wu J, Xiao J, Zhang Z, Zhao Y, Jin Z (2014). Systematic study on G-protein couple receptor prototypes: did they really evolve from prokaryotic genes?. IET Syst Biol.

[101] Cao J, Huang S, Qian J, Huang J, Jin L, Su Z (2009). Evolution of the class C GPCR Venus flytrap modules involved positive selected functional divergence. BMC Evol Biol.

[102] Käll L, Krogh A, Sonnhammer ELL (2004). A combined transmembrane topology and signal peptide prediction method. J Mol Biol.

