# Supplementary material for: Mapping the membrane proteome of anaerobic gut fungi identifies a wealth of carbohydrate binding proteins and transporters
Source: Microb Cell Fact. 2016 Dec 20;15:212. doi: 10.1186/s12934-016-0611-7 (PMC5168858; doi:10.1186/s12934-016-0611-7)
Supplement: Supplementary file 1 — Additional file 1: Figure S1. Putative functions of fungal transporters based on transporter classification data base (TCDB) analysis, showing the mode of transport. 1868 fungal transporter components from three gut fungal strains were sorted based on TCDB homology using a stringent 70% coverage criterion. The mode of transport is indicated. Total number of transcripts encoding putative transporter components in Neocallimastix: 826 transcripts; Anaeromyces: 554 transcripts; Piromyces: 488 transcripts. [file 12934_2016_611_MOESM1_ESM.pdf]

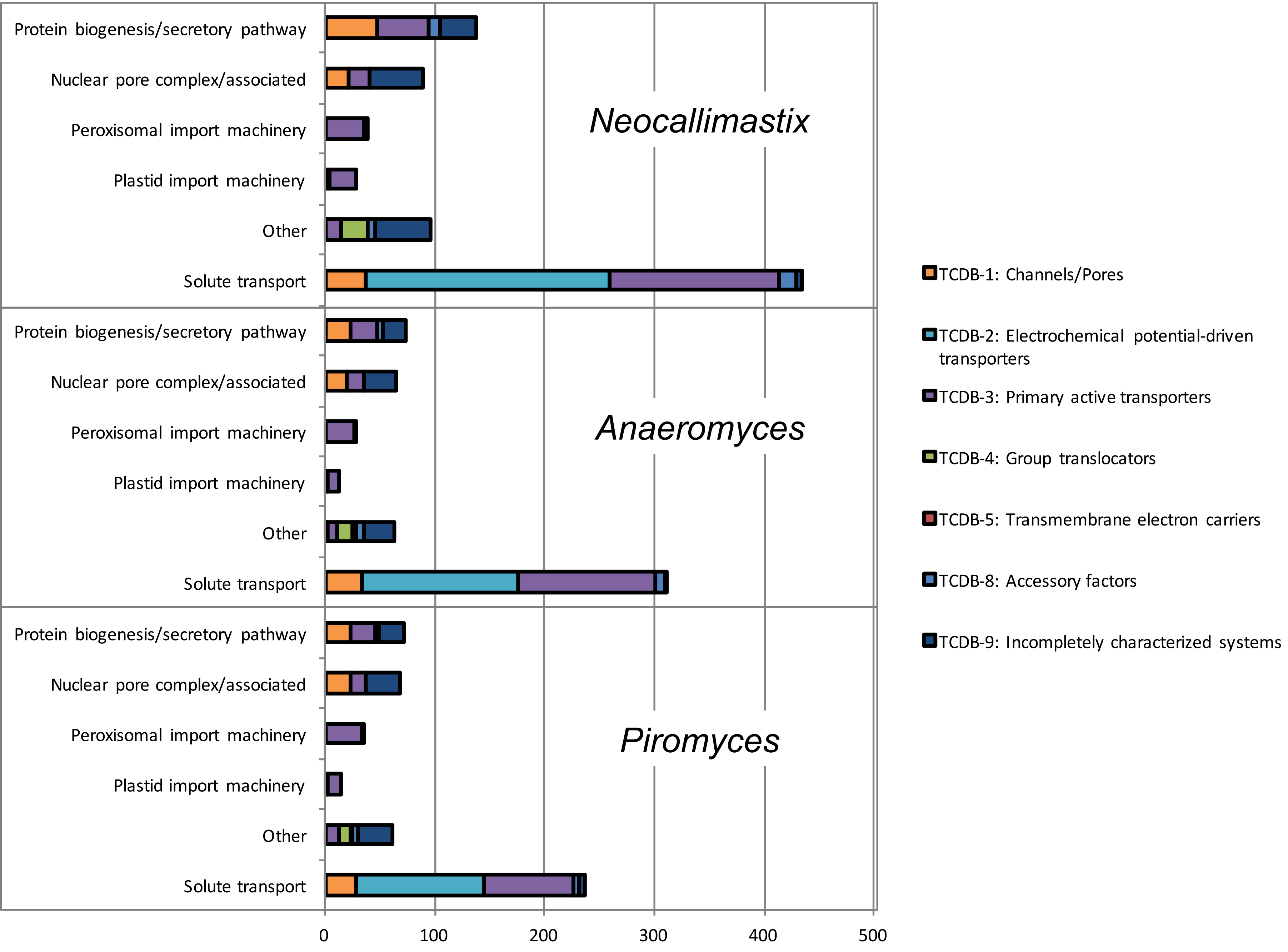
Neocallimastix
Anaeromyces
Piromyces
Protein biogenesis/secretory pathway
Nuclear pore complex/associated
Peroxisomal import machinery
Plastid import machinery
Other
Solute transport
0
100
200
300
400
500
TCDB-1: Channels/Pores
TCDB-2: Electrochemical potential-driven transporters
TCDB-3: Primary active transporters
TCDB-4: Group translocators
TCDB-5: Transmembrane electron carriers
TCDB-8: Accessory factors
TCDB-9: Incompletely characterized systems
