# Supplementary material for: Mapping the membrane proteome of anaerobic gut fungi identifies a wealth of carbohydrate binding proteins and transporters
Source: Microb Cell Fact. 2016 Dec 20;15:212. doi: 10.1186/s12934-016-0611-7 (PMC5168858; doi:10.1186/s12934-016-0611-7)

Figure S2a

Domain architecture of class C GPCRs in *Neocallimastix californiae (Nc)*

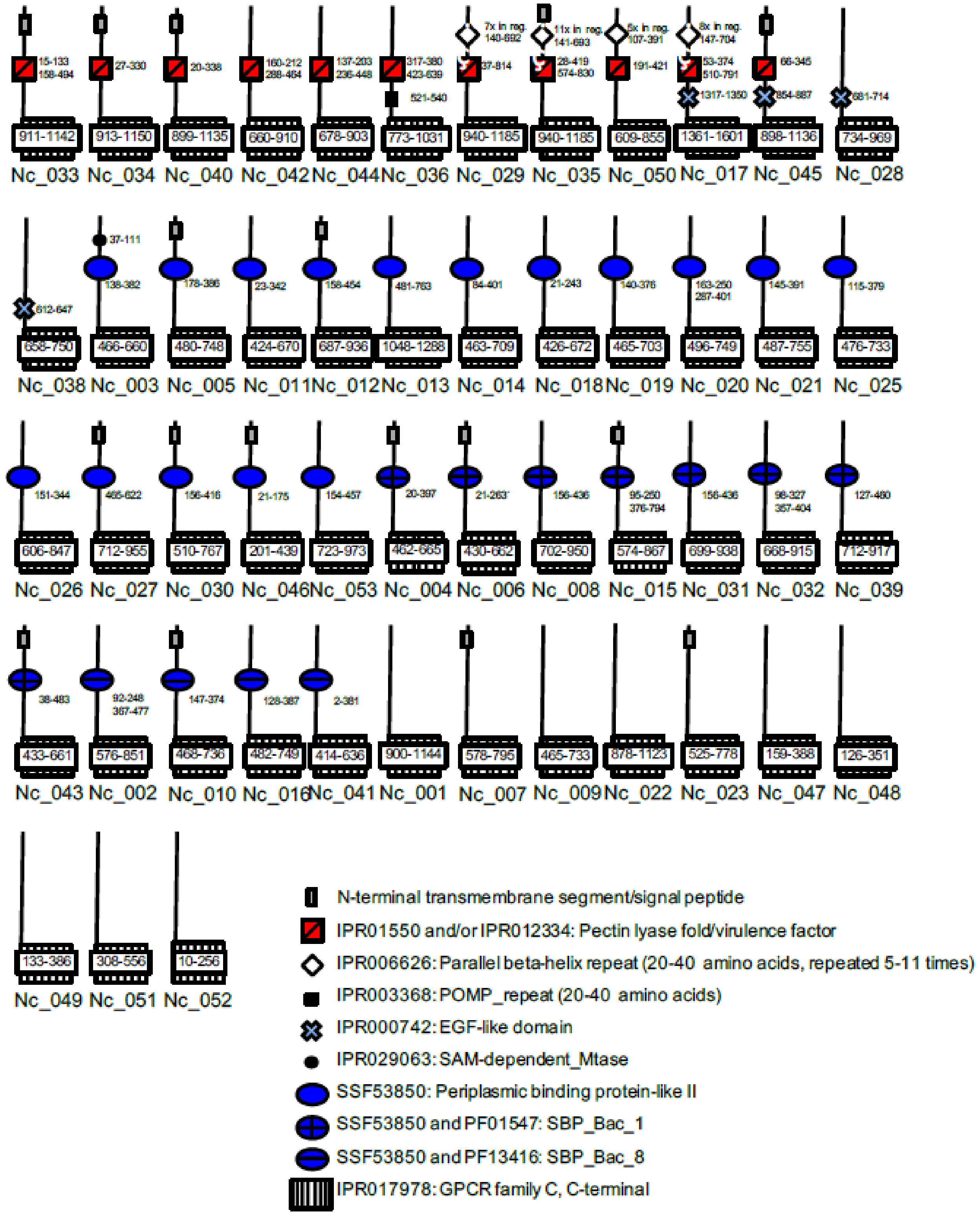

Figure S2b

Domain architecture of class C GPCRs in *Anaeomyces robustus (Ar)*

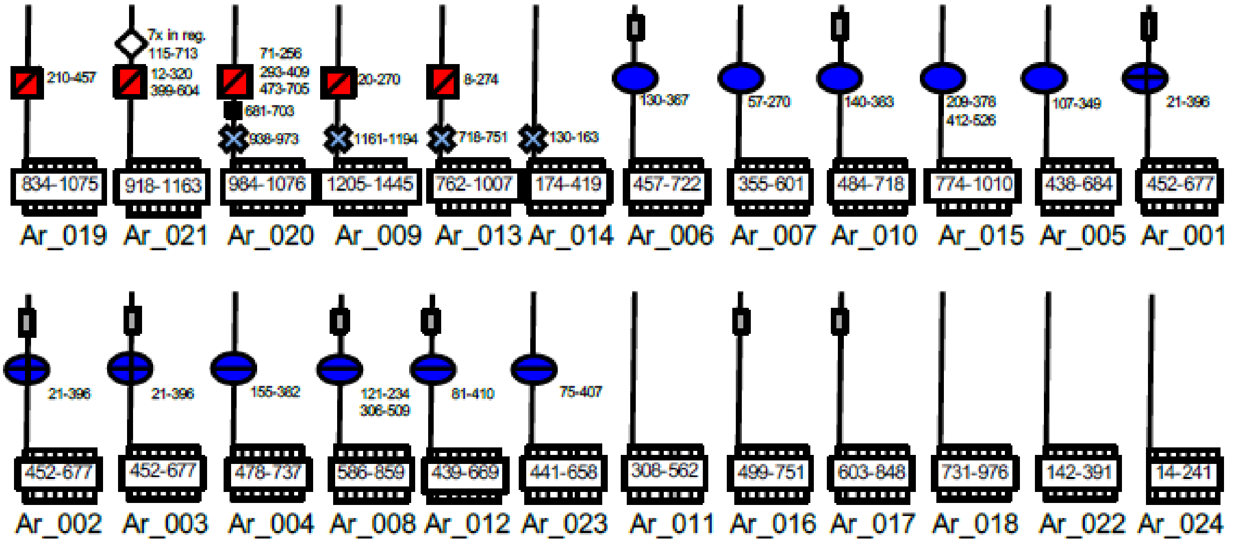

Domain architecture of class C GPCRs in *Piromyces finnis (Pf)*

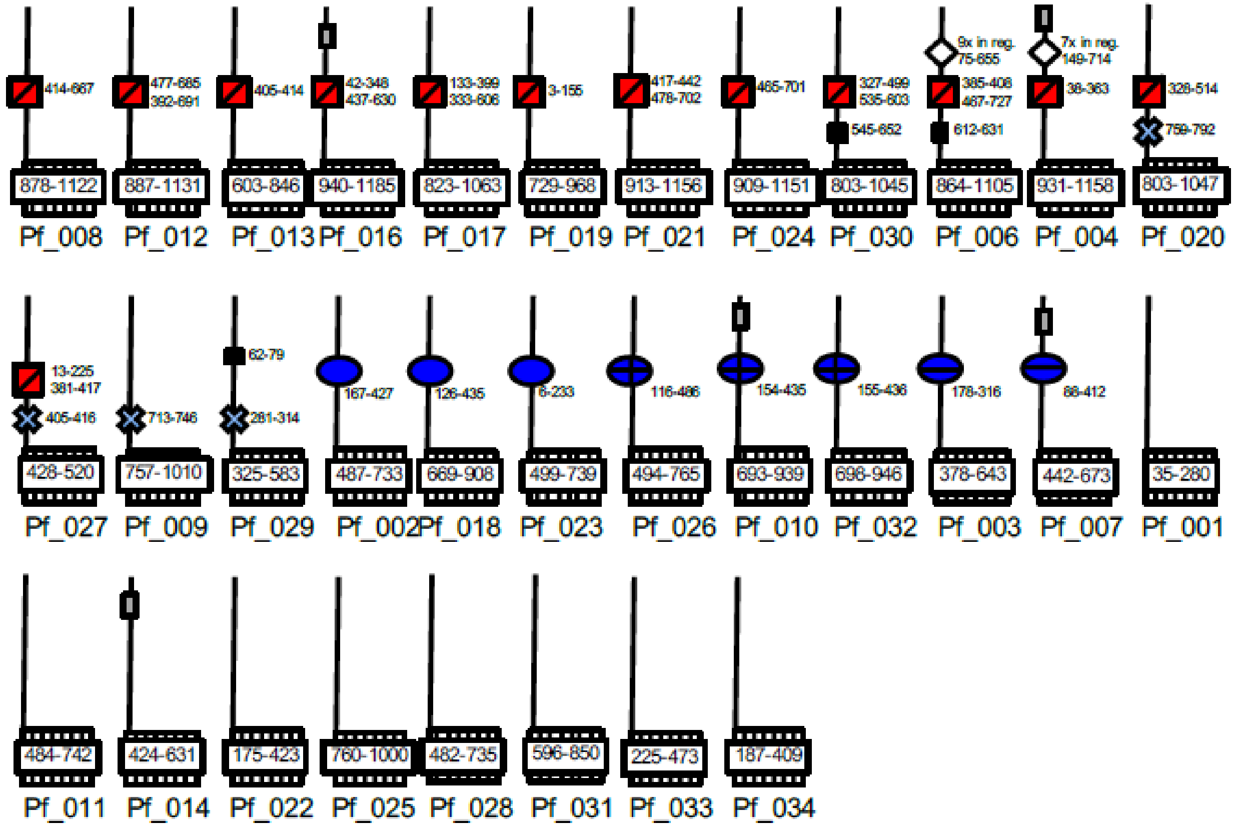

Supplement: Supplementary file 2 — Additional file 2: Figure S2. Detailed domain architecture of gut fungal class C GPCRs as determined using the InterProScan tool. All class C GPCRs are predicted to have a long amino-terminal domain and seven carboxy-terminal transmembrane helices. The amino-terminal domain shows great variability. The predicted positions of the domains on the polypeptide chains are indicated. [file 12934_2016_611_MOESM2_ESM.pdf]
